# Utilizing network pharmacology and experimental validation to explore the potential molecular mechanisms of BanXia-YiYiRen in treating insomnia

**DOI:** 10.1080/21655979.2022.2026862

**Published:** 2022-01-22

**Authors:** Liang Wang, Peng Wang, Yingfan Chen, Chen Li, Xuelin Wang, Yin Zhang, Shaodan Li, Minghui Yang

**Affiliations:** a Chinese PLA Medical School, Beijing, People’s Republic of China; b Department of Traditional Chinese Medicine, Chinese PLA General Hospital, Beijing, People’s Republic of China

**Keywords:** BanXia-YiYiRen, network pharmacology, molecular docking, experimental verification, insomnia, mechanism of action, 5-HT, serotonergic pathway

## Abstract

BanXia-YiYiRen (Pinellia Ternata and Coix Seed, BX-YYR) has been clinically proven to be an effective Chinese medicine compatible with the treatment of insomnia. However, the underlying mechanism of BX-YYR against insomnia remains unclear. This study aimed to explore the pharmacological mechanisms of BX-YYR in treating insomnia based on network pharmacology and experimental validation. The drug-disease targets were obtained using publicly available databases. The analysis revealed 21 active compounds and 101 potential targets of BX-YYR from the pharmacological database of Chinese medicine system and analysis platform (TCMSP) and 1020 related targets of insomnia from the GeneCards and Online Mendelian Inheritance in Man (OMIM) databases. Furthermore, 38 common targets of BX-YYR against insomnia were identified, and these common targets were used to construct a protein–protein interaction (PPI) network. The visual PPI network was constructed by Cytoscape software. The top three genes from PPI according to degree value are FOS, AKT1, and CASP3. Gene Ontology (GO) and Kyoto Encyclopedia of Genes and Genomes (KEGG) pathway enrichment were applied to reveal the potential targets and signaling pathways involved in BX-YYR against insomnia, especially the serotonergic pathway. In addition, molecular docking revealed that baicalein, beta-sitosterol, and stigmasterol displayed strong binding to AKT1, FOS, PRKCA, and VEGFA. Experimental study found that BX-YYR against insomnia might play a role in improving sleep by modulating the serotonergic pathway. In summary, our findings revealed the underlying mechanism of BX-YYR against insomnia and provided an objective basis for further experimental study and clinical application.

## 1. Introduction

Insomnia is characterized by difficulty falling asleep and/or difficulty maintaining sleep, leading to dissatisfaction of sleep time or quality, which seriously damages the patient’s physical and mental health [1]. It is one of the common mental and psychological problems in the social population, and it has attracted increasing attention from society [2]. With the development of the national economic level, the increase in social work pressure and the acceleration of life rhythm changes, the incidence of insomnia in the population shows an increasing trend. At present, the clinical treatment of insomnia is mainly based on sedative and tranquilizing drugs, which easily form dependence and produce side effects in long-term use. However, traditional Chinese medicine (TCM) has certain characteristics and advantages in treating insomnia [3].

The combination of BX-YYR is derived from the formula for insomnia in the ‘Yellow Emperor’s Classic of Internal Medicine’ – Pinellia Rice Decoction, which has a wide range of clinical applications and the effect of improving insomnia symptoms. A study confirmed the good sedative-hypnotic effect of Panicum semifolium soup [4]. Insomnia is related to the dynamic transformation of centrally located neurons as well as neurotransmitters, and numerous studies have found that 4-chloro-DL-phenylalanine (PCPA)-induced insomnia rats have abnormal hippocampal 5-HT and its receptor mechanism. Chinese herbal medicine or acupuncture may improve insomnia by regulating 5-HT1A and 5-HT2A gene expression in the hippocampus [5–7].

Although the combination of BX-YYR and other drugs against insomnia has good efficacy in clinical practice, the specific mechanism of BX-YYR against insomnia at the cellular, molecular and genetic levels is not clear. In recent years, the novel network pharmacology of Chinese herbal medicine has brought together multidisciplinary thinking and theories from traditional pharmacology, bioinformatics and network biology [8]. Network pharmacology can reveal the bioactive components and action mechanism of TCM at the systemic level and reflect the multicomponent and multitarget action relationship of TCM, which is consistent with the holistic concept and evidence-based treatment in TCM theory [9]. Molecular docking is mainly used to obtain sTABLE small molecule (ligand) and target (receptor) complex structures through protein structural domain resolution, intermolecular interaction relationships, and binding during enzyme-substrate catalysis to determine the mechanism of drug action, which is the theoretical basis for new drug design and reflects important application prospects in screening potential and effective active compound small molecules in TCM [10].

**Figure 1.**
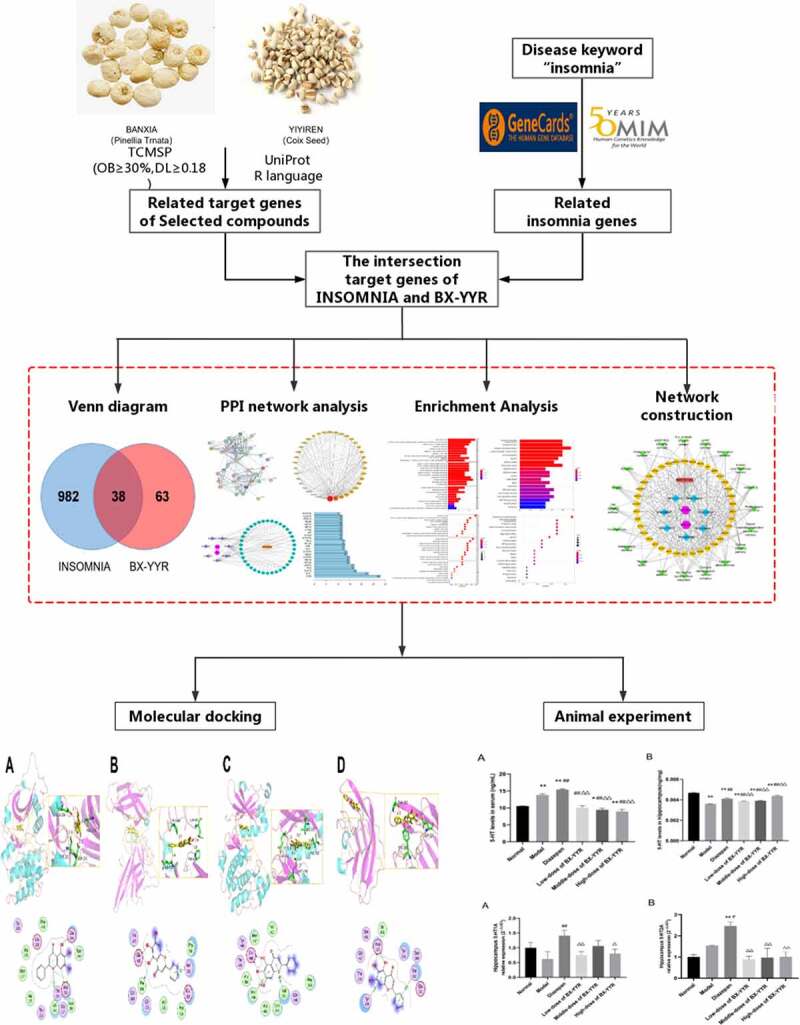
The overall flowchart of this study.

Therefore, the present study was designed to determine the potential mechanisms of major active compounds in BX-YYR in treating insomnia by utilizing network pharmacology and experimental validation. Our research may provide evidence to support the clinical use of BX-YYR against insomnia, help screen effective active compounds of BX-YYR, improve the formulation of BX-YYR, and provide a reliable reference for exploring the pharmacological mechanism of other Chinese medicines treating insomnia (Figure 1).

## 2. Materials and methods

### 2.1. Network pharmacology

#### 2.1.1. Screening for active compounds from BX-YYR

All chemical components of the BX-YYR were collected from the TCMSP databases (http://lsp.nwu.edu.cn/tcmsp.php). We screened the Chinese herbal medicine BX-YYR compounds based on absorption, distribution, metabolism and excretion. In the TCMSP databases, a pharmacokinetic information retrieval filter was used to retrieve bioactive compounds for further analysis under the conditions of oral bioavailability (OB) ≥ 30% and drug-likelihood (DL) ≥ 0.18 [11].

#### 2.1.2. Screening for possible targets from BX-YYR

The TCMSP database was utilized for screening active compounds and corresponding potential therapeutic targets of BX-YYR. The UniProt database (https://www.UniProt.org/) was also used to obtain unique corresponding gene names and UniProt IDs of drugs or disease-related targets [12]. This species was selected as ‘Homo sapiens’, and the names were normalized and entered into an Excel sheet for collation.

#### 2.1.3. Predicting the possible targets of insomnia

Insomnia target screening was performed by searching two databases, the GeneCards database (http://www.genecards.org/) and the OMIM database (http://www.ncbi.nlm.nih.gov/omim) [13], for the keyword ‘insomnia’ [14]. We merged the gene screening results from the two databases. Duplicated targets were removed to harvest the final insomnia target sets.

#### 2.1.4. Analysis of PPI network construction for compound-disease overlapped targets

We used the Venn online tool (http://www.bioinformation.com.cn/) to screen the common targets of BX-YYR against insomnia. The PPI network [15] was constructed by entering the drug-disease common targets into the String V11 database [16] (https://string-db.org/cgi/input.pl) with the species limited to ‘Homo sapiens’ and a confidence score >0.4, which covered almost all functional interactions between the expressed proteins [17]. Cytoscape 3.8.0 software was used to adjust and construct the PPI networks based on the degree values [18,19]. Topology analysis and MCODE analysis for core gene screening used the Network Analyzer tool in Cystoscape [20,21].

#### 2.1.5. GO and KEGG pathway enrichment analysis

We performed GO functional annotation [22] (biological process (BP), molecular function

(MF), cell component (CC)) and KEGG pathway enrichment analysis [23] and used the R 4.0.3 clusterProfiler package [24] to identify the biological processes and signaling pathways involved in BX-YYR in treating insomnia. *P* < 0.05 was considered to identify the enriched terms.

#### 2.1.6. Construction of active component-disease-target-pathway networks

A visual network was constructed using Cytoscape software to reflect the complex relationships between active compounds, disease, common target, and top pathways based on KEGG pathway enrichment analysis. Nodes present the compounds, targets, and pathways, and edges indicate the interactions between pathways, targets, and components that are potentially included in BX-YYR against insomnia.

### 2.2. Molecular docking

#### 2.2.1. Preparation of small molecules from drug components

The component-disease-target network diagram constructed by Cytoscape was topologically analyzed and screened to identify the top three components (baicalein, beta-sitosterol, and stigmasterol) for molecular docking (PubChem database (https://pubchem.ncbi.nlm.nih.gov/) query MOL2 format) and imported into Schrodinger software to build a database, which was saved as a database of molecularly docked ligand molecules by hydrogenation, structure optimization, and energy minimization [25].

#### 2.2.2. Preparation of target protein structures

The protein targets (AKT1, FOS, PRKCA, and VEGFA) were screened by topological and clustering analyses, and their crystal structures (PDB ID: 4GV1, 1A02, 4RA4, 5DN2) were downloaded from the Protein Data Bank (https://www.rcsb.org/structure/). The protein structures were processed in the Maestro 11.9 platform, and the proteins were treated with Schrodinger’s Protein Preparation Wizard [26] to remove crystalline water, add missing hydrogen atoms, repair missing bond information, repair missing peptides, and finally optimize the protein for energy minimization and geometric structure [27].

#### 2.2.3. Visualization of molecular docking

olecular docking was processed and optimized by virtual screening, which was completed by the Glide module in Schrödinger Maestro software. The receptors were preprocessed, optimized and minimized (constrained minimization using the OPLS3e force field). All compounds were prepared according to the default settings of the LigPre module. When screening in the Glide module, we imported the prepared receptors to specify the appropriate positions in receptor grid generation. The original ligand of the protein was selected as the centroid of the 10 Å box [28]. First, the original ligands were docked again to confirm the feasibility of the docking method selection, analyze its mode of action, and obtain the interaction of each target residue (hydrogen bonding, π-π interaction, hydrophobic interaction, etc.), and then compare the compounds score the docking situation and infer whether the screened components have a certain activity.

### 2.3. Animal experiment

#### 2.3.1. Experimental animals

All procedures for animal experiments were carried out under the approval and guidance of the Ethics Committee of the Experimental Animal Center of the PLA General Hospital. Wistar male rats, SPF grade, body weight (160 ± 10) g, for a total of 36 rats, were purchased from Spelford (Beijing) Biotechnology Ltd., animal certificate number: SCXK (Jing) 2019–0010. During the entire experiment, the experimental rats received standard laboratory food and adapted an ambient temperature of 25 ± 2°C, relative humidity (50 ± 5%) and a 12/12-hour light-dark cycle for 1 week.

#### 2.3.2. Insomnia model preparation and experimental grouping

A rat model of insomnia induced by intraperitoneal injection with PCPA once daily for 2 days (Sigma, USA, product lot number 1,003,164,450, 300 mg/kg) was prepared according to the relevant literature [7,29]. At 28 hours after the second intraperitoneal injection of PCPA, it was observed that the circadian rhythm of the insomnia model rats was lost, the daytime activity continued, the excitability and aggression increased, the production of urine and feces increased, the stool was gray–white, the appetite decreased, and the fur was dull and lacking luster. These indicated that the model was successful. According to the random number TABLE method, the insomnia model rats were divided into: normal group, model group, diazepam group, low-dose of BX-YYR, middle-dose of BX-YYR group and high-dose of BX-YYR group, each group 6 only. Six rats in the normal group were injected intraperitoneally with the same volume of weakly alkaline 0.9% sodium chloride.

#### 2.3.3. Interventions and sampling

The same volume of weakly alkaline 0.9% sodium chloride injection was given by gavage in the nondrug group. After the equivalent dose of adult rat body surface area conversion, the diazepam group was intragastrically administered 0.0525 mg/ml diazepam aqueous solution per kg rat. The TCM treatment group included the Chinese herbal medicine low-dose group, Chinese herbal medicine medium-dose group, and Chinese herbal medicine high-dose group, and the Chinese herbal medicine consisted of 10 g BX and 20 g YYR, which were gavaged according to doses containing 1.575 g/kg, 3.15 g/kg, and 6.3 g/kg raw Chinese herbal medicine, respectively. After 7 days of gavage in each group, the rats were anesthetized with sodium pentobarbital (300 mg/kg) intraperitoneally. After blood collection from the abdominal aorta, the hippocampus of each group was dissected on ice, immediately frozen in liquid nitrogen, and finally stored at −80°C for testing.

#### 2.3.4. Animal behavior testing

On the 7th day, the open field experiment was performed on the second day after gavage. Video equipment equipped with an open field box (100 cm×100 cm×45 cm) was used for recording for 5 minutes. Behavioral analysis software was used to analyze the total movement distance (cm), the stay time in the center grid (s) and proportion of the stationary time of the central grid (%). During the experiment, each rat adapted for 5 minutes first, kept the environment quiet throughout the whole process, cleaned up the rat excrement in time after the test of each rat and wiped the open field box with 75% alcohol.

#### 2.3.5. ELISA and real-time PCR assay

ELISA: We used ELISA to detect the 5-HT content in the serum and hippocampus. A rat serotonin (5-HT) ELISA kit (SG3178-96 T, SinoGene, China) and microplate reader (Bio–Rad 680, USA) were used. The main steps were shown in Table 1.

**Table 1.** The main steps of ELISA

Number	Main steps	Specific operation
1	Collect blood/hippocampus	Blood: Blood was collected from the abdominal aorta in a sterile state, rested at 4°C for 1 h. Hippocampus: The hippocampus tissues were removed in a sterile state, placed in a sterile EP tube, and quickly placed in −80 degrees refrigerator for use.
2	Serum extraction /Tissue homogenate	Serum: Centrifuge at 3500 r/min for 15 min, take the serum for use. Hippocampus: Weigh 10 mg tissue, ground with liquid nitrogen, add 90ulPBS, centrifuge at 5000 r/min for 10 min, take the supernatant for use.
3	ELISA kit instructions	Detect 5-HT content of serum and hippocampus: Adding samples, adding enzyme, incubating, liquid preparation, washing, color development, termination, determination.

Real-time PCR: Primers and probes were designed specifically for the target genes of 5-HT1A and 5-HT2A using PrimerBlast (NCBI, Bethesda, Maryland, USA) purchased from Sangon Biotech (Shanghai, China). The main steps were shown in Table 2. The Ct values of each sample were corrected to obtain ΔCt values using ACTB as the internal reference gene. The data were calculated by the 2-deltadeltaCt (2-ΔΔCt) method. ΔΔCt = each group (Ct target gene-CtACTB)-control group (Ct target gene-CtGAPDH), 2-ΔΔCt = gene expression of each group/gene expression of control group.

**Table 2.** The main steps of real-time PCR

Number	Main steps	Specific operation
1	Total RNA extraction	Rat brain hippocampal tissue was broken with TRIzol, centrifuged at low temperature 12,000 r/min for 15 min, the supernatant was aspirated, and total RNA was extracted by sequentially adding chloroform and isopropanol.
2	DNase I treatment	37°C water bath for 30 minutes; 65°C water bath for 10 minutes to inactivate DNase I
3	mRNA reverse transcription	Mix and centrifuge; annealing at 25°C for 5 min, extension at 42°C for 60 min, and inactivation at 70°C. 5-HT1A mRNA: upstream primer 5′-tgttgctcatgctggttctc-3′, downstream primer 5′-ccgacgaagttcctaagctg-3′; 5-HT2a mRNA: upstream primer 5′-tcatcatggcagtgtcccta-3′, downstream primer 5′-acaggcatgacaaggaaacc-3′.
4	Real-time PCR	Initial denaturation(95°C, 10 min, 1 cycles); Denaturation(95°C, 20s, 40cycles); Annealing(60°C, 30s, 40cycles); Dissociation(95°C, 15s, 1 cycles; 60°C, 30s, 1 cycles; 95°C, 15s, 1 cycles)

### 2.4. Statistical methods

SPSS 22.0 statistical software and GraphPad Prism 8.0.2 software were used to statistically analyze the data, and the data are expressed as the mean ± standard deviation $\left(\bar{x}\pm \;s\right)$. The data did not conform to a normal distribution using a nonparametric test; the data conformed to a normal distribution with equal variance using an independent t test, the data conformed to a normal distribution with unequal variance using an approximate t test, and the difference was considered statistically significant at *P* < 0.05.

## 3. Results

### 3.1. Screening of active compounds in BX-YYR

The results of the study showed that 21 active ingredients in BX-YYR met the requirements of OB and DL. Among them, BX contained 13 active ingredients, and YYR contained 9 active ingredients. The two shared 1 identical ingredient, stigmasterol (MOL000449) (Table 3).

**Table 3.** Some active ingredients of BX-YYR

Mol ID	Molecule Name	OB (%)	DL	Origin
MOL001755	24-Ethylcholest-4-en-3-one	36.08	0.76	BX
MOL002670	Cavidine	35.64	0.81	BX
MOL002714	baicalein	33.52	0.21	BX
MOL002776	Baicalin	40.12	0.75	BX
MOL000358	beta-sitosterol	36.91	0.75	BX
MOL005030	gondoic acid	30.70	0.20	BX
MOL000519	coniferin	31.11	0.32	BX
MOL006936	10,13-eicosadienoic	39.99	0.2	BX
MOL006937	12,13-epoxy-9-hydroxynonadeca-7,10-dienoic acid	42.15	0.24	BX
MOL006957	(3S,6S)-3-(benzyl)-6-(4-hydroxybenzyl)piperazine-2,5-quinone	46.89	0.27	BX
MOL003578	Cycloartenol	38.69	0.78	BX
MOL006967	beta-D-Ribofuranoside, xanthine-9	44.72	0.21	BX
MOL000449	Stigmasterol	43.83	0.76	BX, YYR
MOL001323	Sitosterol alpha1	43.28	0.78	YYR
MOL001494	Mandenol	42.00	0.19	YYR
MOL002372	(6Z,10E,14E,18E)-2,6,10,15,19,23-hexamethyltetracosa-2,6,10,14,18,22-hexaene	33.55	0.42	YYR
MOL002882	[(2 R)-2,3-dihydroxypropyl] (Z)-octadec-9-enoate	34.13	0.30	YYR
MOL000359	sitosterol	36.91	0.75	YYR
MOL008118	Coixenolide	32.40	0.43	YYR
MOL008121	2-Monoolein	34.23	0.29	YYR
MOL000953	CLR	37.87	0.68	YYR

### 3.2. Target prediction results

**Figure 2.**
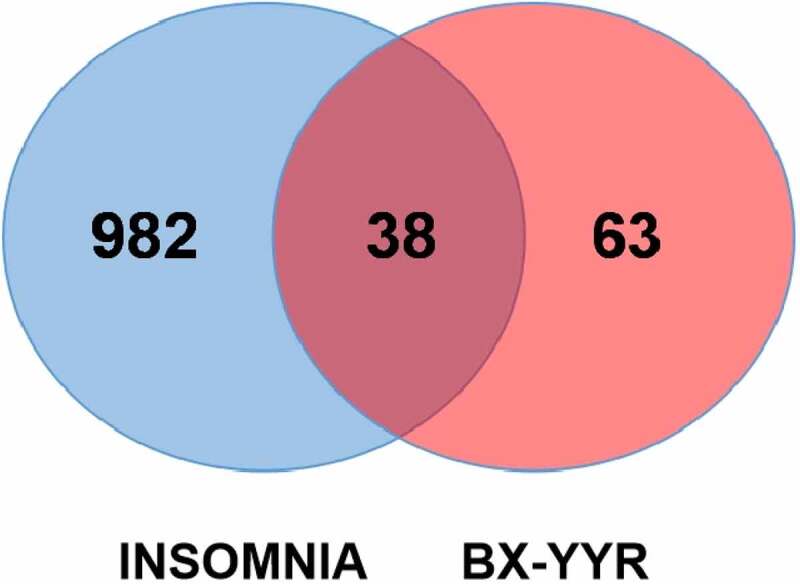
Venn diagram of the target genes between BX-YYR and insomnia.

Using the UniProt database and R language, the protein targets corresponding to the active ingredients of BX-YYR were searched on the TCMSP data platform and converted into the corresponding human gene name. A total of 101 targets were obtained gene. We searched the Genecards and OMIM databases with ‘Insomnia’ to obtain 2567 disease target genes. After screening, combination and deduplication, 1020 disease target genes were obtained. The target genes of BX-YYR were mapped to genes related to insomnia, and 38 common target genes were obtained. The proteins corresponding to 38 target genes were used as the targets of BX-YYR against insomnia, and the active ingredients of BX-YYR against the insomnia target map were established (Figure 2), where the light blue circle represents insomnia, the red circle represents the active ingredient targets of BX-YYR, and the intersecting ellipse represents the key targets shared by BX-YYR against insomnia.

### 3.3. PPI network construction

**Figure 3.**
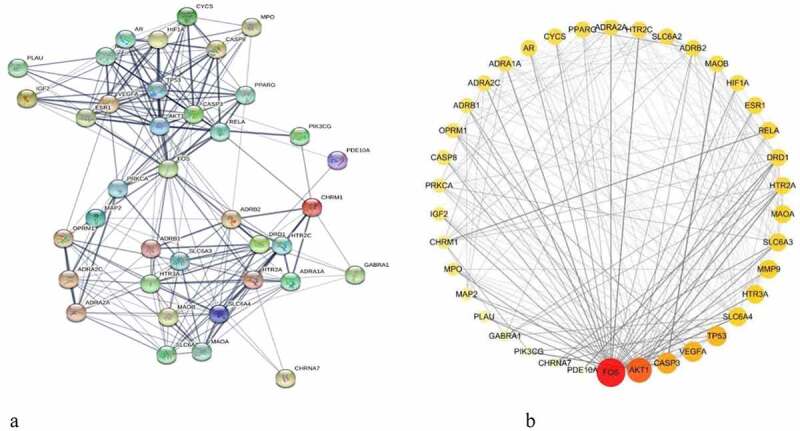
Protein–protein interaction (PPI) network and node network diagram in Cytoscape. (a) PPI network diagram of the common targets between BX-YYR and insomnia. (b) PPI network diagram of BX-YYR-insomnia common targets by Cytoscape.

The PPI network was constructed by inputting 38 drug-disease common targets into the STRING database and setting the biological species to ‘Homo sapiens’. There were 38 nodes and 224 edges in this network, and the average degree values from the STRING database were shown in Figure 3(a). The PPI network diagram of Figure 3(b) was plotted by Cytoscape software. The color and size of the nodes in Figure 3 were adjusted according to the degree value; the larger the node, the redder the color, and the line from thick to thin indicated the edge betweenness from large to small.

### 3.4. Topology and cluster analysis

**Figure 4.**
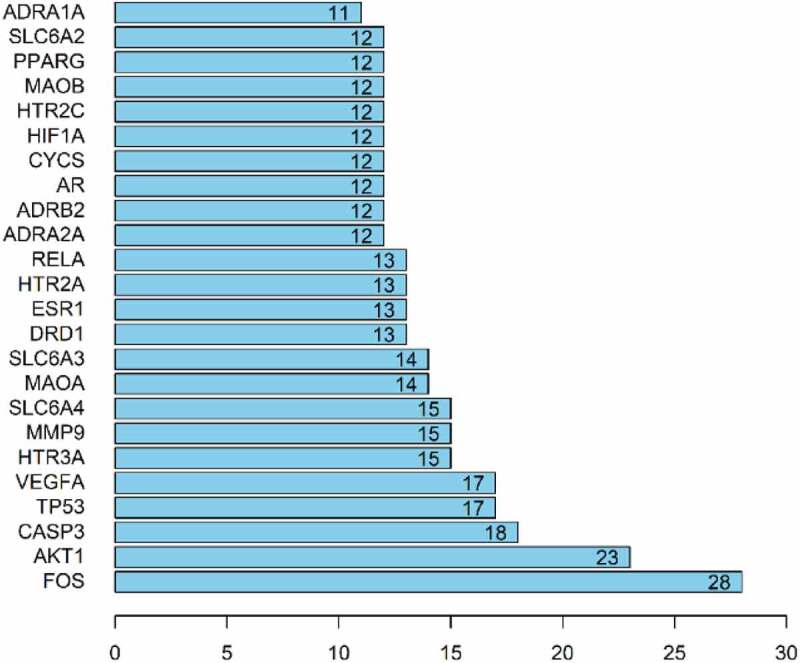
Topological analysis diagram of the core target genes.

The PPI network was imported into Cystoscape 3.8.0, topological analysis was performed by the Network Analyzer tool, genes with scores greater than the average score were selected as key targets by degree sorting, and a total of 24 key targets were selected. As shown in Figure 4.

Core genes were screened by MCODE cluster analysis. The constructed PPI network was imported into Cytoscape 3.8.0, and the MCODE module was opened for gene cluster analysis and core target screening. Two gene clusters and 2 core genes were obtained, and the core genes were *PRKCA* and *VEGFA*. The specific results are shown in the cluster analysis information table. As shown in Table 4.

**Table 4.** Clustering analysis diagram of the core target genes between BX-YYR and insomnia

Cluster	Network	Nodes	Edges	Node IDs
1	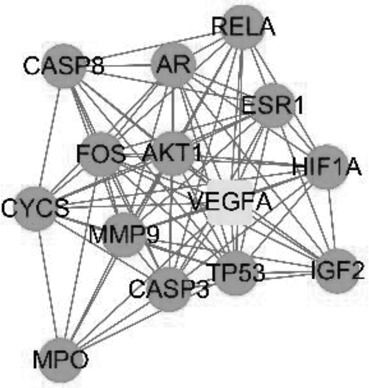	14	80	*CASP8, CASP3, CYCS, TP53, VEGFA, MPO, HIF1A, FOS, ESR1, IGF2, RELA, AR, MMP9, AKT1*
2	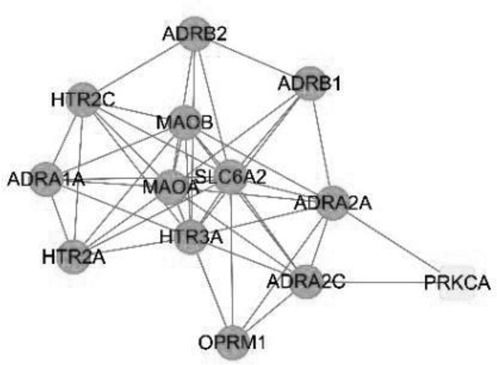	13	46	*ADRA2A, HTR2A, ADRB2, MAOB, HTR3A, MAOA, HTR2C, OPRM1, PRKCA, ADRA2C, SLC6A2, ADRA1A, ADRB1*

between BX-YYR and insomnia.

### 3.5. Constructing an ingredient-disease-target network and screening key components

**Figure 5.**
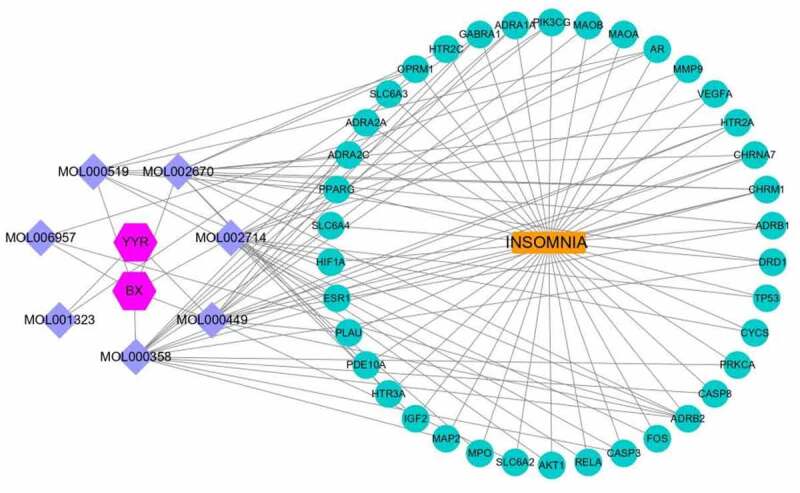
Component-disease-target network between BX-YYR and insomnia.

To better understand and study the complex relationships between the active ingredients of BX-YYR, insomnia diseases and their corresponding targets, a component-disease-target network diagram was constructed based on the incorporated components, therapeutic diseases and targets, and Cytoscape 3.8.0 was imported to draw the network diagram and perform topological analysis. As shown in Figure 5. The components were ranked by degree value, and a higher degree value indicated that the components were more important (Table 5).

**Table 5.** Key component information table for the component-disease-target network diagram between BX-YYR and insomnia

MOL ID	Name	Average Shortest Path Length	Betweenness Centrality	Closeness Centrality	Degree
MOL000358	beta-sitosterol	2.212766	0.066625	0.451923	15
MOL000449	Stigmasterol	2.212766	0.101333	0.451923	15
MOL002714	baicalein	2.297872	0.058374	0.435185	14
MOL002670	Cavidine	2.382979	0.039254	0.419643	12
MOL000519	coniferin	2.553191	0.017069	0.391667	8

### 3.6. GO function enrichment analysis and KEGG signaling pathway

**Figure 6.**
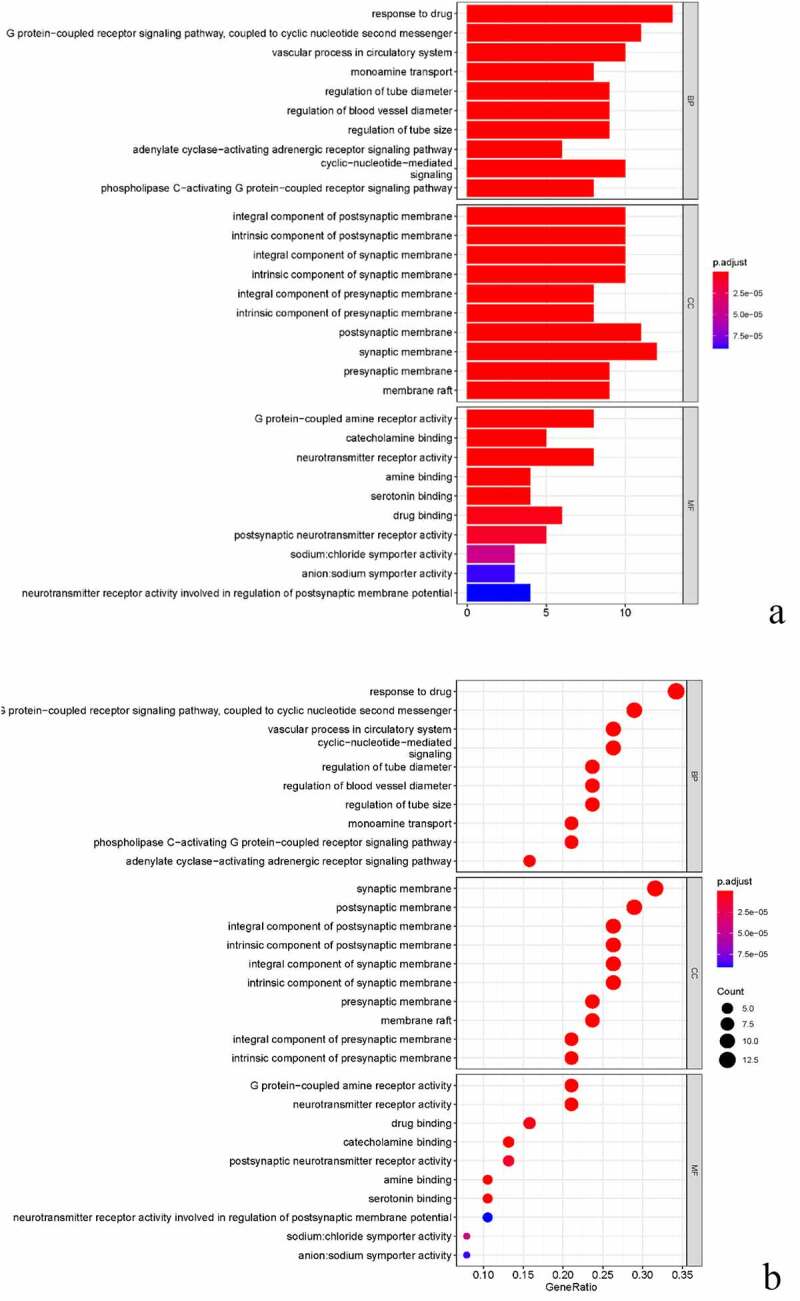
GO analysis of common targets. (a) Histogram of GO enrichment analysis between BX-YYR and insomnia predicted. (b) Top 10 bubble diagram of GO enrichment analysis between BX-YYR and insomnia predicted target top10.

We enriched the biological process (BP), molecular function (MF) and cell component (CC) of GO for the common targets of BX-YYR and insomnia, filtered the items with corrected *P* value <0.05 by referring to the STRING database, and enriched a total of 897 biological processes, 84 molecular function-related and 39 cell component-related, which were enriched, mainly involving the regulation of G protein-coupled neurotransmitter receptor activity, adrenoceptor activity, serotonin binding, catecholamine binding, RNA polymerase II transcription factor binding and anion transport activity. Using R 4.0.3 software, the ClusterProfiler, Enrichplot and Ggplot2 packages were installed and referenced for bar and bubble plotting, and the top 10 entries for BP, CC, MF enrichment of GO were listed (Figure 6(a,b).

**Figure 7.**
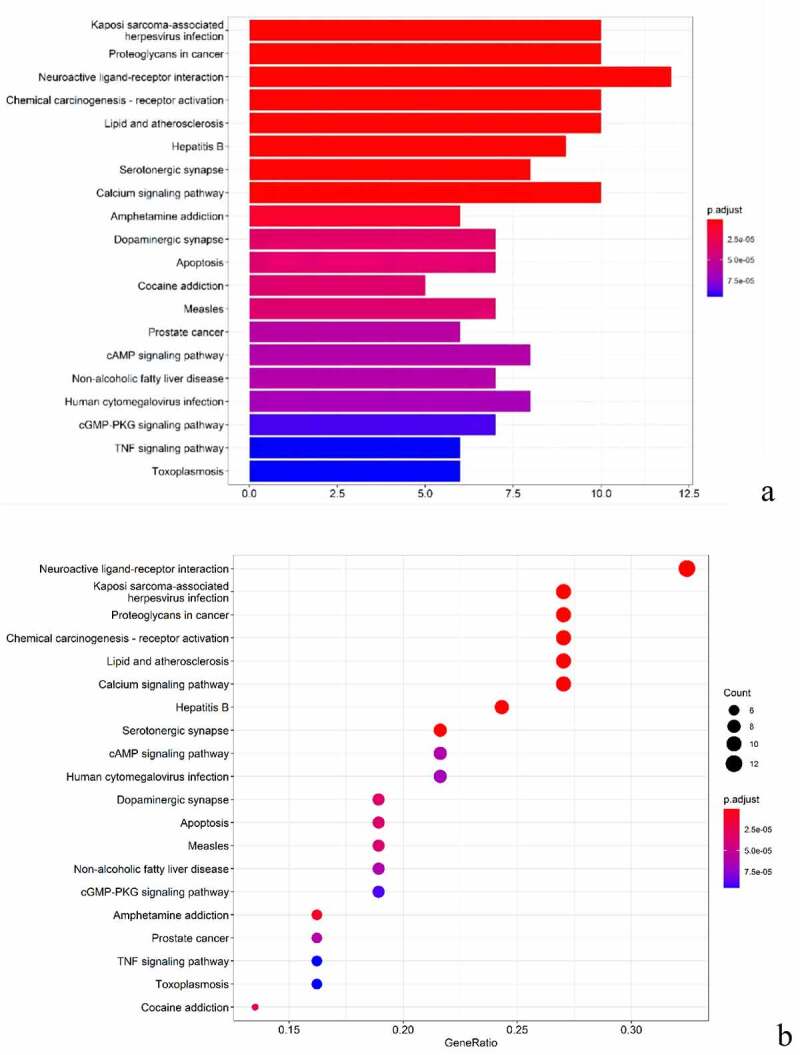
KEGG analysis of common targets. (a) Histogram of KEGG pathways between BX-YYR and insomnia predicted the top 10 targets. (b) Bubble diagram of the top 10 KEGG pathways between BX-YYR and insomnia predicted target top10.

The KEGG pathway enrichment analysis was performed on the common targets of BX-YYR and insomnia, and the items with corrected *P* value <0.05 were screened by referring to the STRING database, and a total of 109 signaling pathways were enriched, involving neuroactive, apoptotic, immune function, amino acid metabolism and other signaling pathways, mainly including neuroactive ligand–receptor interactions, serotonin synapses, calcium signaling pathway, amphetamine addiction, dopaminergic synapses, apoptosis, cAMP signaling pathway, cGMP-PKG signaling pathway, TNF pathway, and other signaling pathways. After installing and referencing the clusterProfiler package using R 4.0.3, histograms and bubble plots can be drawn (Figure 7(a,b)).

### 3.7. Constructing ingredient-disease-target-pathway network

**Figure 8.**
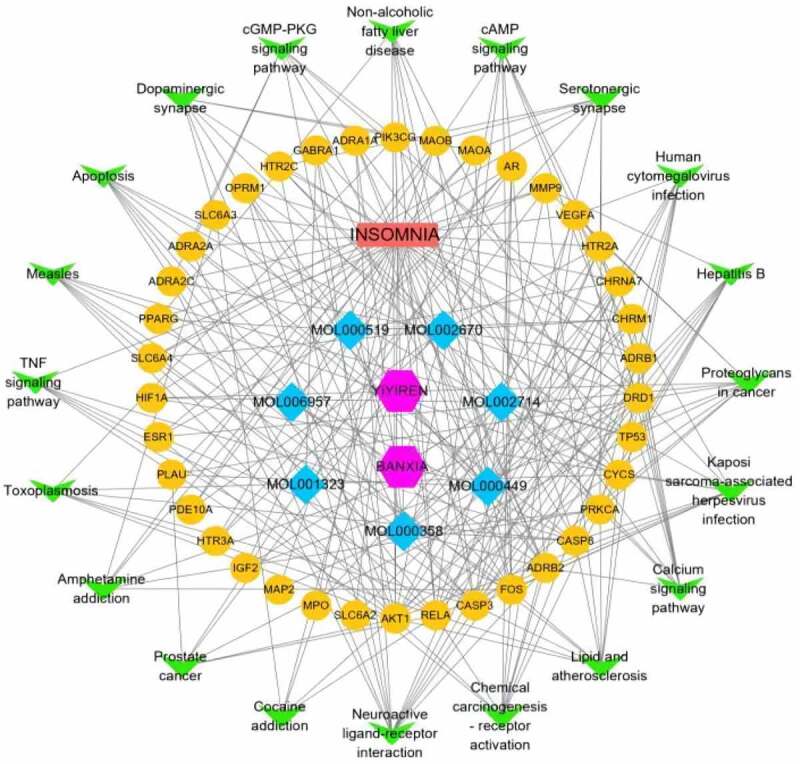
Constructing a component-disease-target-pathway network for the treatment of insomnia with BX-YYR. Green triangles represent pathways, yellow dots represent coacting targets, blue diamonds represent core active components, and pink hexagons represent semisummer and coix kernels, respectively.

The component-disease-target-pathway network file was imported into Cytoscape 3.8.0 for pathway network mapping to more visually show the multicomponent, multitarget and multipathway characteristics in the process of treating insomnia with the active ingredients of the Chinese herbal medicine BX-YYR. As shown in Figure 8, blue is the compound, yellow is the target of Chinese herbal medicine made for disease, green is the top 20 most significant KEGG pathways, red is insomnia, and purple is the Chinese herbal medicine BX-YYR.

### 3.8. Molecular docking

#### 3.8.1. Screening results

Molecular docking of baicalein, beta-sitosterol, and stigmasterol with four target proteins (AKT1, FOS, PRKCA, VEGFA) was performed. The molecular docking results showed that the binding energies of the compounds and the four target proteins were less than −5 kcal/mol and that there was a strong binding effect. The lower binding energy of the compounds to the targets indicated better binding. Overall, the absolute value of the binding energy scored by FOS to small molecules is lower than that of several other targets, mainly because the altered target proteins have no original ligands and the target activity pocket is unclear, which poses a greater challenge to molecular docking. The molecular docking results are shown in Table 6.

**Table 6.** Results of molecular docking between small drug molecules and target proteins

Name	Compound Structure	Target	Binding Energy (kcal/mol)	Combination Type
Baicalein	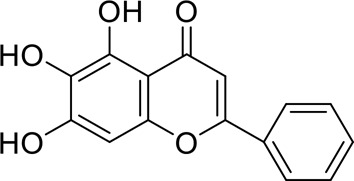	AKT1	−7.01	Hydrogen bonds, Hydrophobic interactive, π-stacking
beta-Sitosterol	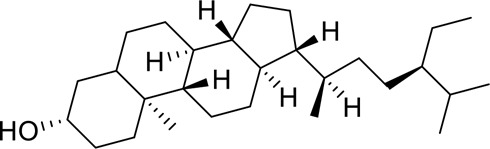	AKT1	−6.18	Hydrogen bonds, Hydrophobic interactive, π-stacking
Stigmasterol	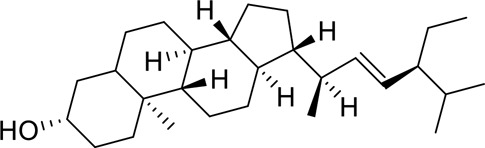	AKT1	−6.02	Hydrogen bonds, Hydrophobic interactive, π-stacking
Baicalein	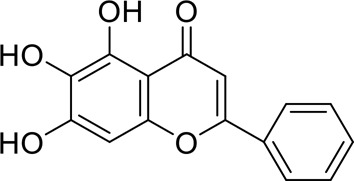	FOS	−6.65	Hydrogen bonds, Hydrophobic interactive, π-stacking
beta-Sitosterol	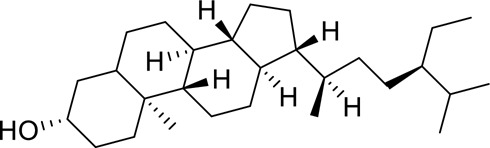	FOS	−5.79	Hydrogen bonds, Hydrophobic interactive, π-stacking
Stigmasterol	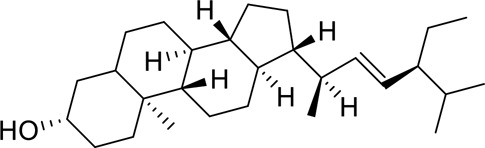	FOS	−5.56	Hydrogen bonds, Hydrophobic interactive, π-stacking
Baicalein	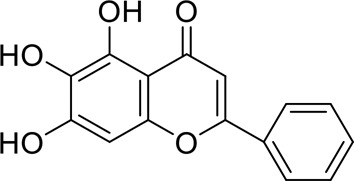	PRKCA	−7.65	Hydrogen bonds, Hydrophobic interactive, π-stacking
beta-Sitosterol	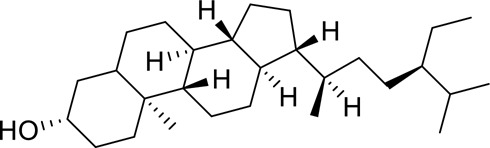	PRKCA	−6.92	Hydrogen bonds, Hydrophobic interactive, π-stacking
Stigmasterol	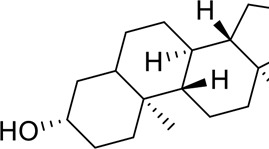	PRKCA	−6.85	Hydrogen bonds, Hydrophobic interactive, π-stacking
Baicalein	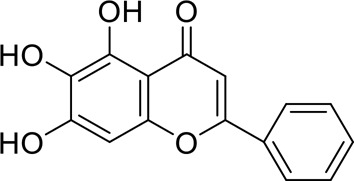	VEGFA	−7.16	Hydrogen bonds, Hydrophobic interactive, π-stacking
beta-Sitosterol	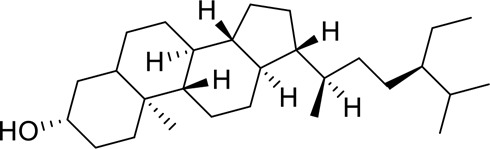	VEGFA	−6.19	Hydrogen bonds, Hydrophobic interactive, πstacking
Stigmasterol	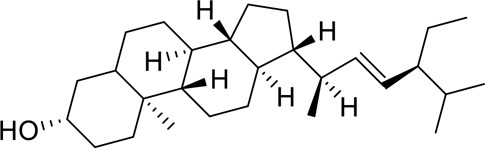	VEGFA	−6.13	Hydrogen bonds, Hydrophobic interactive, π-stacking

Note: Binding energy fuction [28]: $\begin{array}{c} \Delta G_{bind}=C_{lipo-lipo}\sum^{f}(r_{lr})+C_{hbond-neut-neut}\sum^{g}(\Delta r)h(\Delta \alpha )+C_{hbond-neut-charged}\sum^{g}(\Delta r)h(\Delta \alpha )+\\ C_{hbond-charged-charged}\sum^{g}(\Delta r)h(\Delta \alpha )+C_{max-metal-ion}\sum^{f}(r_{lm})+C_{rotb}H_{rotb}+\\ C_{polar-phob}V_{polar-phob}+C_{coul}E_{coul}+C_{vdW}E_{vdW}+Solvationterms \end{array}$

#### 3.8.2. Protein interaction analysis

**Figure 9.**
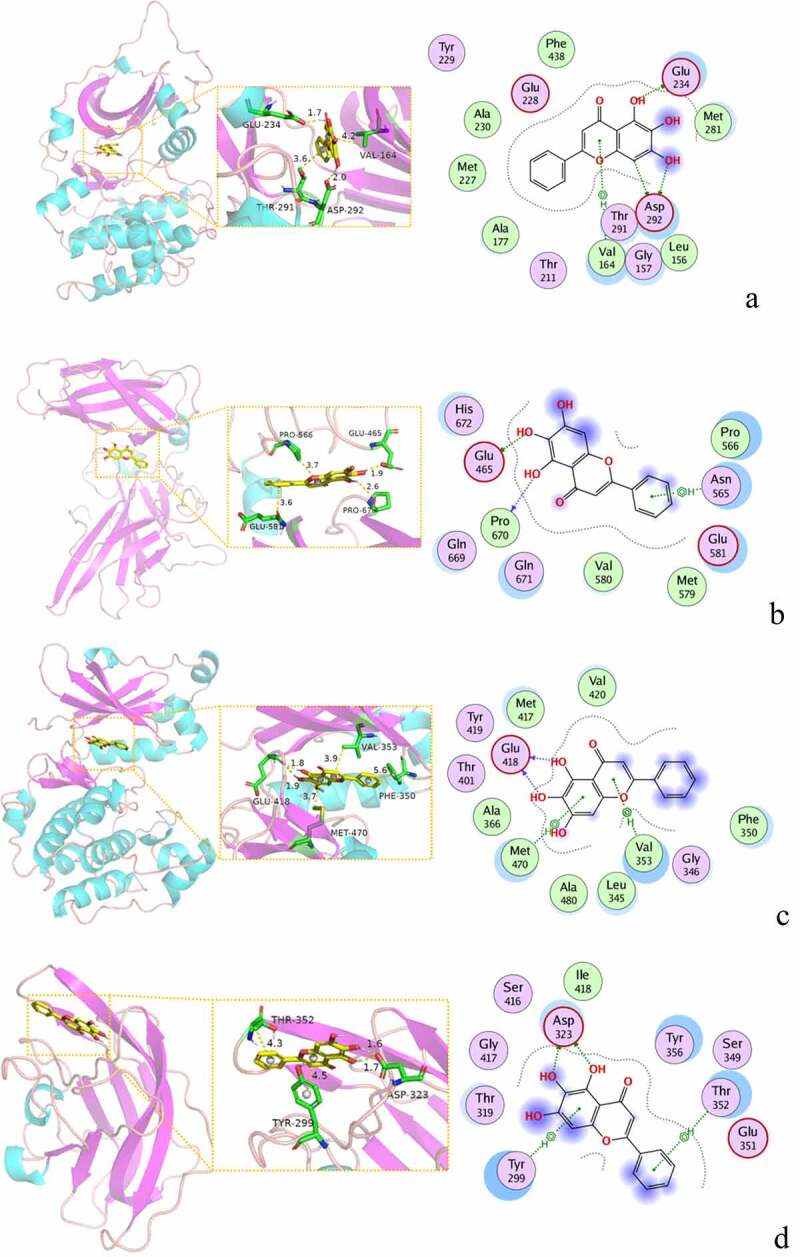
3D molecular docking diagrams of active ingredients and targets. (a) The binding mode of the AKT1 complex with baicalein. (b) The binding mode of the FOS complex with baicalein. (c) The binding mode of the PRKCA complex with baicalein. (d) The binding mode of the VEGFA complex with baicalein.

The complexes formed by the compounds and proteins after docking were visualized using PyMOL 2.1 software to obtain the binding pattern of the compounds and proteins. According to the binding pattern, the amino acid residues bound by the compounds and protein pockets can be clearly seen, e.g., the active amino acid residues bound by baicalein to AKT1 are GLU-234, ASP-292, THR-baicalein belongs to flavonoids and has more reactive groups, forming hydrogen bonds with amino acid reactive groups, such as hydroxyl groups forming stronger hydrogen bonds with the reactive groups of GLU-234 and ASP-292 with an average distance of 1.85 Å, which is smaller than the traditional hydrogen bond of 3.5 Å. The binding is stronger and has an important role in stabilizing small molecule ligands. In addition, baicalein also has a strong hydrophobic interaction with the active pocket of the protein; for example, its benzene ring is able to form π-π conjugation interactions with hydrophobic amino acids VAL-164 and THR-291. Molecular docking is visualized in Figure 9(a-d).

### 3.9. Animal experiments

#### 3.9.1. Open field experiment

**Figure 10.**
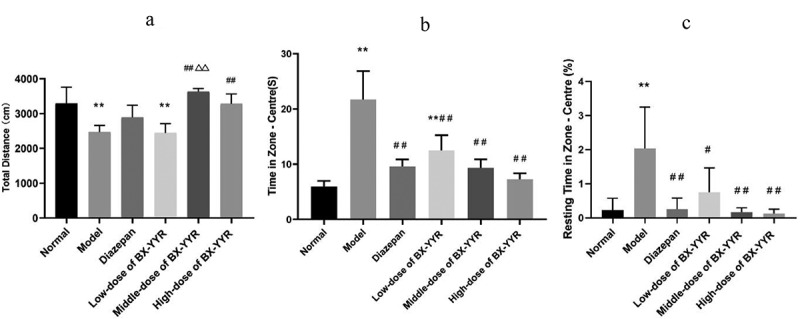
Open field experiment. (a) Total movement distance. (b) Time in Zone-Center. (c) Resting Time in Zone-Center (%). (Note: compared with the normal group, *P < 0.05, **P < 0.01; compared with the model control group, ^#^*P* < 0.05, ^##^*P < 0.01*; compared with the diazepam group, ^Δ^*P*<0.05, ^ΔΔ^*P<0.01*.).

**Figure 11.**
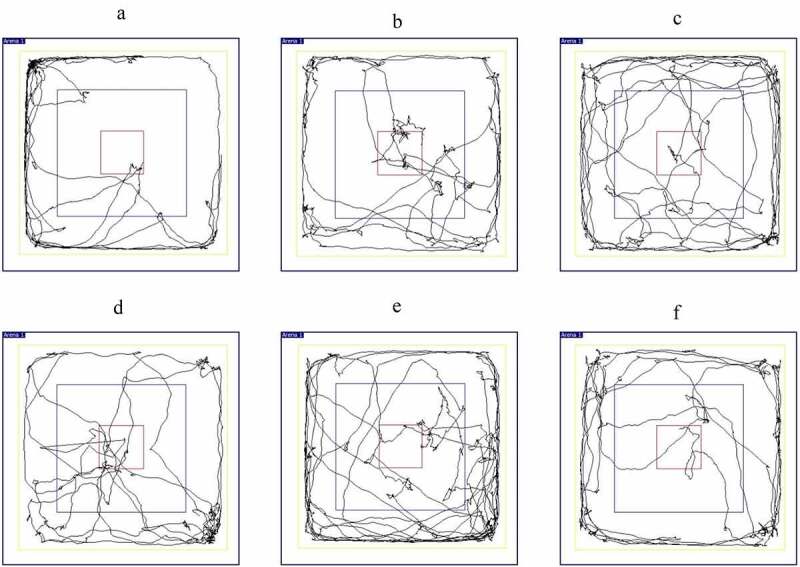
The rat trajectory diagram of each group in the open field experiment. (a) Normal group. (b) Model group. (c) Diazepam group. (d) Low dose of BX-YYR group. (e) Medium dose of BX-YYR group. (f) High dose of BX-YYR group.

Compared with the normal group, the total movement distance of rats in the model group and low-dose groups was extremely significantly reduced (*P* < 0.01, *P* < 0.01). Compared with the model group, the total activity distance of the medium- and high-dose groups increased significantly (*P* < 0.01, *P* < 0.01). Compared with the diazepam group, the total activity distance of the medium-dose groups increased significantly (*P* < 0.01). As shown in Figure 10(a) and 11.

Compared with the normal group, the time spent in the zone center of the low-dose groups was increased (*P* < 0.05). Compared with the model group, the time spent in the zone center of the high-dose groups was reduced (*P* < 0.05). As shown in Figure 10(b).

Compared with the normal group, the resting time in the zone center of the model group was extremely significantly increased (*P* < 0.01). Compared with the model group, the resting time in the zone center of diazepam and the low-, medium- and high-dose groups was reduced (*P* < 0.01, *P* < 0.05, *P* < 0.01, *P* < 0.01). As shown in Figure 10(c).

#### 3.9.2. ELISA detection of 5-HT level in serum and hippocampus

**Figure 12.**
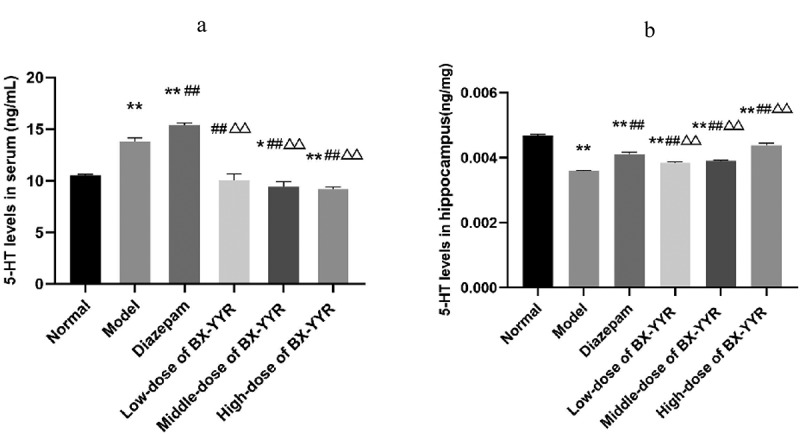
Differential level of 5-HT in serum and hippocampal. (a) 5-HT levels in Serum. (b) 5-HT levels in hippocampal. (Note: compared with the normal group, *P < 0.05, **P < 0.01; compared with the model control group, ^#^*P* < 0.05, ^##^*P* < 0.01; compared with the diazepam group, ^Δ^*P*<0.05, ^ΔΔ^*P*<0.01.).

Compared with the normal group, serum 5-HT levels were significantly increased in the model control group and diazepam group rats (*P* < 0.01, *P* < 0.01), while serum 5-HT levels were decreased in the Chinese herbal medicine medium- and high-dose groups (*P* < 0.05, *P* < 0.01). Compared with the model control group, the serum 5-HT level of rats in the diazepam group was significantly increased (*P* < 0.01), and the serum 5-HT level of rats in the Chinese herbal medicine low-, medium- and high-dose groups was significantly decreased (*P* < 0.01, *P* < 0.01, *P* < 0.01). Compared with the diazepam group, the serum 5-HT levels of rats in the low-, medium- and high-dose groups of Chinese herbal medicine were significantly reduced (*P* < 0.01, *P* < 0.01, *P* < 0.01). For details, see Figure 12(a).

Compared with the normal group, the hippocampal 5-HT levels of rats in the model control group, the diazepam group, and the low-, medium- and high-dose groups of Chinese herbal medicines were significantly reduced (*P* < 0.01, *P* < 0.01, *P* < 0.01, *P* < 0.01, *P* < 0.01, *P* < 0.01). Compared with the model control group, the hippocampal 5-HT levels of rats in the diazepam group and the low-, medium- and high-dose groups treated with Chinese herbal medicine were significantly increased (*P* < 0.01, *P* < 0.01, *P* < 0.01, *P* < 0.01, *P* < 0.01). Compared with the diazepam group, the hippocampal 5-HT levels of rats in the low- and medium-dose groups treated with Chinese herbal medicine were decreased (*P* < 0.01, *P* < 0.01). As shown in Figure 12(b).

#### 3.9.3. Detection of 5HT1A mRNA and 5HT2B mRNA by real-time PCR

**Figure 13.**
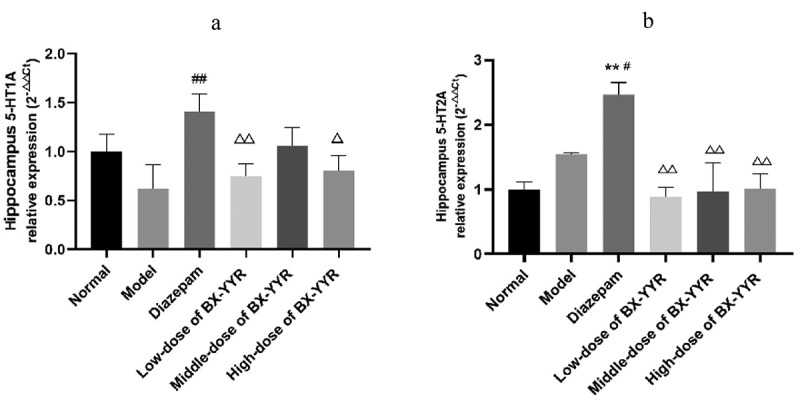
Differential expression of 5-HT1A and 5-HT2A mRNA in the hippocampus. (a) 5-HT1A mRNA expression in hippocampal. (b) 5-HT1A mRNA expression in hippocampal. (Note: compared with the normal group, *P < 0.05, **P < 0.01; compared with the model control group, ^#^*P* < 0.05, ^##^*P < 0.01*; compared with the diazepam group, ^Δ^*P*<0.05, ^ΔΔ^*P<0.01*.).

Compared with the normal group, hippocampal 5HT1A mRNA expression was decreased in the model control group and the low- and high-dose groups treated with Chinese herbal medicine (all *P* > 0.05), while hippocampal 5HT1A mRNA expression was increased in the diazepam group and the medium-dose group treated with Chinese herbal medicine (all *P* > 0.05). Compared with the model control group, hippocampal 5HT1A mRNA expression was significantly increased in the diazepam group (*P* < 0.01), and hippocampal 5HT1A mRNA expression was significantly increased in the low-, medium- and high-dose groups of Chinese herbal medicines (all *P* > 0.05). Hippocampal 5HT1A mRNA expression was decreased in the low-, medium- and high-dose groups treated with the Chinese herbal medicine compared with the diazepam group (*P < 0.01, P > 0.05, P < 0.01*). As shown in Figure 13(a).

Compared with the normal group, the expression of 5HT2A mRNA was elevated in the model control group and diazepam group (*P > 0.05, P < 0.01*), and the difference in hippocampal 5HT2A mRNA expression in the low-, medium- and high-dose groups of Chinese herbal medicines was smaller (all *P > 0.05*). Hippocampal 5HT2A mRNA expression was significantly higher in the diazepam group than in the model control group (*P* < 0.01). Compared with the diazepam group, hippocampal 5HT2A mRNA expression was significantly lower in the hippocampus of rats in the low-, medium- and high-dose groups treated with Chinese herbal medicine (*P* < 0.01, *P* < 0.01, *P* < 0.01). As shown in Figure 13(b).

## 4. Discussion

As insomnia becomes more common, Chinese medicine is gradually gaining attention from clinicians as an alternative option for insomnia [30]. TCM has a long history of relieving insomnia symptoms and improving sleep quality [31], and healing and calming therapy is an important treatment for insomnia. Considering the complex mechanism of action of the BX-YYR herbal compound, a more complete and systematic study of the challenges of the BX-YYR combination for insomnia is necessary to further guide future clinical and research directions. The compatibility of BX and YYR reflects one warming and one cold, complementing each other, nourishing the spleen and stomach, while the spleen and stomach are the hub for lifting and lowering, the stomach qi decreases to smoothness, the stomach qi decreases, the spleen qi rises, the clear yang increases the turbidity and the yin decreases, and phlegm The dampness is eliminated, the two combined to treat insomnia play a role in reconciling the upper and lower yin and yang [32]. Literature studies have found that the extraction method using ethanol as a solvent helps us to extract more molecules of other biologically active substances from Chinese herbal medicines [33] and can analyze the dynamic changes of its main small molecule compounds in different periods [34]. Studies on the pharmacological properties of Pinellia ternata [35] show that the ethanol extract of Pinellia ternata has sedative, hypnotic and anticonvulsant activities [36] and can enhance the hypnosis induced by pentobarbital sodium [37], which may be related to the GABAergic system. A pharmacological study of Coix Seed found that Coix Seed extract can inhibit the arousal response of EEG to external auditory stimulation [38] and has anti-inflammatory, antioxidant and sedative effects [39,40].

The results of the network pharmacology of this study showed that there were 21 effective compounds for BX-YYR, and the active ingredient with the highest oral utilization was MOL006957 [(3S,6S)-3-(benzyl)-6-(4-hydroxybenzyl)piperazine-2,5-quinone], at 46.89%. In addition, MOL006967 (beta-D-ribofuranoside, xanthine-9), MOL000449 (stigmasterol), MOL001323 (sitosterol alpha1), and MOL006937 (12,13-epoxy-9- hydroxynonadeca-7,10-dienoic acid) were ranked in the top five for oral utilization, with MOL000449 (stigmasterol) being the active ingredient shared by the drug pair of BX-YYR; thus, presumably, these ingredients play an important pharmacological role in the treatment of insomnia by BX-YYR. There were 101 active ingredient targets corresponding to BX-YYR, among which 38 common targets were related to insomnia. The PPI results show that the key targets for the treatment of insomnia by BX-YYR are FOS, AKT1, CASP3, TP53, VEGFA, etc. These key targets involve 897 BP, 84 MF and 39 CC, mainly including the regulation of G protein-coupled neurotransmitter receptor activity, serotonin binding, catecholamine binding, etc. In addition, they are associated with neuroactive ligand–receptor interactions, serotonin synapses, calcium signaling pathway, amphetamine addiction, dopaminergic synapses, apoptosis, cAMP signaling pathway and other KEGG signaling pathway modulation.

The molecular docking results revealed that baicalein, beta-sitosterol, and stigmasterol have strong binding interactions with four target proteins (AKT1, FOS, PRKCA, and VEGFA) with molecular docking energies less than −5 kcal/mol. Among them, the molecular docking interaction of baicalein with AKT1 improves the stability of the baicalein compound in the AKT1 protein pocket and is predicted to be a potentially active small molecule. As one of the flavonoids in many traditional Chinese medicine extracts, baicalein has pharmacological effects such as sedation and anti-anxiety effects [3]. In the central nervous system (CNS), baicalein exerts a protective effect on neurons, and it effectively improves the pharmacology of sleep activity, which may depend on GABAergic nonbenzodiazepine sites [41]. Liu et al. found that beta-sitosterol can regulate sleep structure by activating the GABA A system [42], while research by Ghias Uddin et al. showed that beta-sitosterol has sedative and anti-inflammatory effects in animal models [43].

Several other compounds, such as beta-sitosterol and stigmasterol, have slightly weaker binding ability to proteins due to their large molecular weight and hydrophobicity but also have multiple interactions and contribute more to stabilizing small molecules, which are also potentially active compounds and small molecules for the treatment of insomnia with BX-YYR in terms of macromolecular protein structural domain resolution. It provides a pharmacological mechanism for the treatment of insomnia by BX-YYR in terms of protein structure domain resolution, interactions between macromolecules, and binding in the enzyme-substrate catalytic process, which is valuable for screening the active ingredients of Chinese herbal medicine for the treatment of insomnia.

Studies have shown that the FOS protein is involved in the normal differentiation, growth, learning, memory and other processes of cells and is more highly expressed in the brain, cortex, hippocampus, and striatum [44]. AKT1 is involved in metabolism, proliferation, growth and regulation of angiogenesis and other processes [45]. The levels and metabolism of various neurotransmitters [46], such as dopamine, 5-HT, and epinephrine, are involved in the onset and maintenance of sleep [47], and melatonin also requires 5-HT to produce and maintain the normal functioning of the sleep cycle [48,49]. Gao Wei et al. found that the IGF-1/PI3K/AKT signaling pathway in the hippocampus of mice after REM sleep deprivation may be involved in the regulation of the expression of proinflammatory factors [50]. The improvement of learning and memory in sleep-deprived rats is related to the decrease in the expression of CASP3 and other proteins [51]. VEGFA, or vascular endothelial growth factor A, is involved in the VEGF signaling pathway, a neurotrophic factor related to angiogenesis and neurogenesis, and affects synaptic plasticity [52]. Zhang Xuan et al. demonstrated that IL-17 could act directly (or indirectly) to inhibit hippocampal neural progenitor cell proliferation after sleep deprivation by activating the p38 MAPK signaling pathway [53]. Studies have confirmed that sleep deprivation activates inflammatory signaling pathways and increases monocyte production of IL-6 and TNF and that the inflammatory response may cause damage to the host [54]. Studies have found significant effects on the expression of genes of the neuroactive ligand–receptor interaction signaling pathway, such as long-term use of sleeping drugs [55]. Liu, Unity et al. found that activation of dopamine D1 receptor (D1R) on neuronal cell membranes enhances neuronal excitatory effects by upregulating cAMP response element binding protein (CREB) through pathways such as PKA and MAPK [56], while the cGMP-PKG signaling pathway plays an important role in regulating neurogenesis and enhancing cell proliferation [57].

In mammals, serotonin is widely known as a neurotransmitter and hormone produced by intestinal chromophores in the gastrointestinal tract and circulating platelets [58]. 5-HT interacts with its receptor family and plays an important role in the central nervous system (CNS) and various organ systems, as well as regulating a variety of cognitive, behavioral, and physiological functions of the body [59], including mood, sleep-wake rhythm, energy balance, gastrointestinal function, and immunity [60]. Studies have shown that 5-HT in the CNS accounts for only 5% of the total body volume [61], and all regions of the CNS express 5-HT receptors in a subtype-specific manner, with many serotonin receptor subtypes, such as 5-HT1A, 5-HT2A, 5-HT1B, 5-HT3 and 5-HT7, involved in immunomodulatory effects [62]. Increased REM sleep is associated with 5-HT neuronal activity, and 5-HT1A receptor activation can increase arousal [63], thus participating in sleep regulation. 5-HT1A receptors stimulate cell proliferation, differentiation, and apoptosis, and they are highly expressed in the hippocampus, internal olfactory cortex, frontal cortex, and median septal nucleus [64]. Studies have reported that 5-HT1A receptors induce apoptosis in CHO cells and that their antagonist binding to 5-HT1A receptors leads to a deinhibitory effect of 5-HT and increases 5-hydroxytryptaminergic neuronal activity [65], enhances cholinergic and glutamatergic transmission, and improves cognitive function [66]. 5-HT2 plays an important role in the regulation of arousal and sleep duration, and 5-HT2A receptors are also significantly expressed in the hippocampus [64]. Sleep deprivation increases the binding capacity of 5-HT2A receptors. Pharmacology confirms that 5-HT2A receptor signaling can promote cortical arousal and thus influence the sleep-improving effects of drugs [67]. Studies have shown that the western drug diltiazem enhances pentobarbital-induced sleep [68], which is associated with the 5-HTergic pathway, including 5-HT1A and 5-HT(2A/2 C) receptors. The results of the present animal experiments suggest that BX-YYR intervenes in hippocampal 5-HT and its receptor expression in PCPA insomnia model rats, possibly by elevating the hippocampal 5-HT content and 5-HT1A expression level and decreasing the 5-HT content and hippocampal 5-HT2A expression level, thus acting as a sleep-wake regulator. This result is consistent with the literature mentioning that PCPA achieves insomnia by depleting 5-HT in the brain, validating the serotonergic pathway predicted in the KEGG pathway for BX-YYR treatment of insomnia.

In summary, this study usednetwork pharmacology and molecular docking methods to systematically explore and verify the potential mechanism of BXYYR in the treatment of insomnia from multiple perspectives for the first time. Finally, the animal experimental verification results show that this method can better explain the network characteristics and the synergistic action principle of Chinese medicine compounds, indicating that BX-YYR is a multicomponent, multi-target and multi-pathway drug in the treatment of insomnia. Next, on the basis of this research, this research group will use Chinese medicine chromatography and mass spectrometry technology analysis methods to verify the data mining results of active ingredients, laying a theoretical and experimental basis for future in-depth exploration of molecular biology, cell experiments and pharmacology.

## 5. Conclusion

By using the method of network pharmacology, we have studied the potential targets of BX-YYR and its underlying mechanism of improving insomnia symptoms, which reflects its characteristics of multiple components, multiple targets, and multiple pathways. FOS, AKT1, CASP3, TP53 and VEGFA may be potential targets of BX-YYR in the treatment of insomnia. According to the enrichment analysis results of GO and KEGG signaling pathways, we found pathways closely related to the pathological process of insomnia, mainly including serotonergic pathways, neuroactive ligand–receptor interactions, calcium signaling pathways, dopaminergic synapses, and apoptotic cell death. Preliminary validation of molecular docking showed that the small molecule compounds (baicalein, beta-sitosterol, stigmasterol) contained in BX-YYR generally have excellent binding affinity for the large molecular target proteins (AKT1, FOS, PRKCA, VEGFA) encoded by the first 4 genes. Experimental validation revealed that the treatment of insomnia with BX-YYR was associated with the regulation of serotonergic signaling pathways, which resulted in improved sleep. Therefore, this study identified that BX-YYR could treat insomnia through directly or indirectly regulating the abovementioned potential targets and pathways. BX-YYR may provide a promising direction for future insomnia therapy, but also relevant experimental studies, which are very important to reveal the exact pharmacological mechanism ofBX-YYR on insomnia, should been carried out in the future study.

## Data Availability

The data used to support the findings of this study are included within the article.

## Highlights

- Baicalein, beta-sitosterol and stigmasterol might be the major active compounds in the treatment of insomnia by BX-YYR.
- The therapeutic effect of BX-YYR against insomnia might be achieved by altering the proteins AKT1, FOS, PRKCA, and VEGFA.
- Animal experiments have verified the mechanism by which BX-YYR improves sleep by regulating the serotonergic signaling pathway.
